# Manufacturing Technology and Properties of Fe/TaC Metal Matrix Composite Coatings Produced on Medium Carbon Steel Using Laser Processing—Preliminary Study on the Single Laser Tracks

**DOI:** 10.3390/ma14185367

**Published:** 2021-09-17

**Authors:** Dariusz Bartkowski

**Affiliations:** Institute of Materials Technology, Faculty of Mechanical Engineering, Poznan University of Technology, Piotrowo 3 Street, 61-138 Poznan, Poland; dariusz.bartkowski@put.poznan.pl; Tel.: +48-616-652-665

**Keywords:** tantalum carbide, laser processing, microstructure, EDS, microhardness

## Abstract

The paper presents study results of Fe/TaC metal matrix composite coatings produced on tool steel using laser processing of TaC pre-coat. The Fe/TaC coatings were produced in two steps. First, a pre-coat in the form of a paste based on tantalum carbide and water glass was made and then applied to the steel substrate. In the second step, the TaC pre-coat was remelted with steel substrate using a diode laser beam with a rated power of 3 kW. A constant scanning speed of the laser beam of 3 m/min and three types of laser beam power: 500 W, 800 W and 1100 W were applied. Tests were carried out on three different thicknesses of the TaC pre-coat: 30 µm, 60 µm and 90 µm. The influence of pre-coat thickness and laser beam power on the microstructure, chemical composition and microhardness were analyzed. A possibility of producing coatings with a characteristic composite structure was found, where the iron from the substrate became the matrix, and the introduced tantalum carbides—the reinforcing phase. It was found that too high power of the laser beam leads to complete melting of the introduced primary TaC particles. It was also found that the use of a thicker TaC pre-coat contributes to microhardness increase.

## 1. Introduction

Laser surface treatment of ferrous and non-ferrous alloys is becoming more and more widespread not only among researchers, but also in industrial space. Production of wear-resistant coatings on steels intended for extreme operating conditions such as soil, stone, rock, oil and in gas or mining industries is becoming more and more common [1,2]. The MMC (metal matrix composition) coatings in which the matrix is nickel-based (NiCrBSi) [3,4] or cobalt-based (Stellite) [2,5] alloys reinforced with hard particles of primary tungsten carbides WC are well known. Much fewer papers describing iron-based composite coatings, apart from a paper focusing on Fe/WC coatings, are available [6,7,8,9,10,11,12,13,14]. There are many methods of making composite coatings. These are, for example, laser cladding technology [1,2,3,4,5,6,7,8,9,11,12,14,15,16,17,18,19,20,21,22,23,24,25,26,27], traditional surfacing technologies [28,29,30,31] or laser processing of pre-coat [32,33]. Regardless of the production method used, it is important to obtain a composite microstructure, where the reinforcing phase will be clearly separated from the metal matrix. It is also important to check the possibility of producing iron-based composite coatings reinforced with other carbides, e.g., TaC—tantalum carbide. Researchers have tried to use this carbide. However, most often, matrices of nickel or cobalt alloys were described. In paper [19] the authors analyzed Ni-based composite coating reinforced with in situ synthesized TaC particles produced on mild steel using laser cladding. Powder mixture of Ni60 alloy powder with (Ta_2_O_5_ + C)-doping was used. The authors focused on microstructure and wear resistance of the TaC/Ni60 coating. It was found that the coating was bonded metallurgically to the substrate and had a homogeneous fine microstructure containing both approximate cubic TaC particle and acicular chromium carbide uniformly dispersed in the dual phase matrix of nickel solid solution. The authors stated that microhardness of TaC/Ni60 composite coating was enhanced in comparison to Ni60 coating, and finally achieved 1100 HV0.3. Wear resistance was reduced fivefold, which is due to the presence of in situ synthesized TaC particles and their good distribution in the coating. Hu et al. [1] focused on Ni-based coating reinforced with Ni_3_Ta-TaC synthesized in-situ on the substrate of the cutter ring using laser cladding. Their premise was to improve its wear resistance. The authors described microstructure and strengthening mechanism of Ni-Ta and Ni-Ta-C coatings. The Ni-Ta coating was mainly composed of γ-Ni, Ni_3_Ta and Ta phases, while the Ni-Ta-C coating was mainly composed of γ-Ni, Ni_3_Ta and TaC phases. The authors found that strengthening phases Ni_3_Ta and TaC contributed to increased hardness. In paper [22] the laser cladding process of nickel-based powders with the addition of nanopowders of tantalum carbide and tungsten carbide with water-based hydroxyethylcellulose as the binder was described. Coatings with additives of TaC nanoparticles at various weight concentrations (5%, 10%, 15%, 20%) were obtained. The authors showed a 4–6-fold decrease in weight loss along with an increase in concentration of nano-additives of tantalum carbide in mechanical tests. In paper [23,24] the authors focused on production of coatings with additive nanoparticles of refractory metals (WC, TaC) using laser cladding process. Comparative endurance tests for coatings containing nano-WC and nano-TaC were conducted. It was found that coatings containing TaC particles were more durable than nano-WC coatings. The authors explained that melting point of TaC (3800 °C) was higher than that of WC (2800 °C). Hence, WC also has a greater tendency to disintegrate into carbon and tungsten and into secondary W_2_C carbides. Wear resistance of coating with TaC additives increases 4-fold as compared to the coating without a nanopowder additive. The same authors [25] also focused on laser cladding of nickel-based powders with TaC nanopowder additives. A comparison of microstructure obtained with the use of standard nickel-based powder and standard nickel-based powder with additives of different concentrations of TaC nanopowder. The authors found an increase in microhardness with reduction of TaC nanoparticle concentration. In paper [18] the authors analyzed the effects of tantalum on microstructure and microhardness of Ni-based (NiCrBSi) coating produced using laser cladding. Through addition of tantalum, fine TaC particles were synthesized in the coating. The amount of primary carbides (M_7_C_3_) and eutectic (γ-Ni + M_23_C_6_) substantially decreased because the formation of TaC particles suppressed their formation. On the one hand, fine TaC particles improved the microhardness of the composite coating, and on the other hand, caused reduced crack susceptibility of the Ni-based composite coating. Tantalum also improved wear resistance of the coating. Yan et al. [17] described TaC/StelliteX-40 composite coatings fabricated on nickel-aluminum bronze substrates using laser cladding. The main purpose was to improve wear and corrosion resistances of this substrate in marine environments. The authors found a uniform distribution of carbides and inter-metallic reinforcements (TaC, Cr_3_C_2_ and Co_3_Ta) in Co-based matrix. It led to improved wear resistance and electrochemical corrosion resistance. A content of 20 wt% TaC contributed to fine reinforcements with isocellular crystals in the surface region and columnar crystals near the substrate area. Metallurgical bonding between the coating and substrate was found. With increasing TaC content in the powders the number of the reinforcements in the laser surface cladding coatings increased gradually, and the particles became larger. The authors continued their studies in paper [3]. The NiCrBSi + Ta coating exhibited higher fracture toughness, and higher abrasive and adhesive wear resistance than the NiCrBSi coating without tantalum.

As in available papers the authors have not dealt with Fe-based coatings reinforced with TaC so far, this paper presents the results of studies on such composite coatings. Preliminary studies have been carried out on single laser tracks. The micro-structure, microhardness and chemical composition were analyzed.

## 2. Materials and Methods

**Figure 1:**
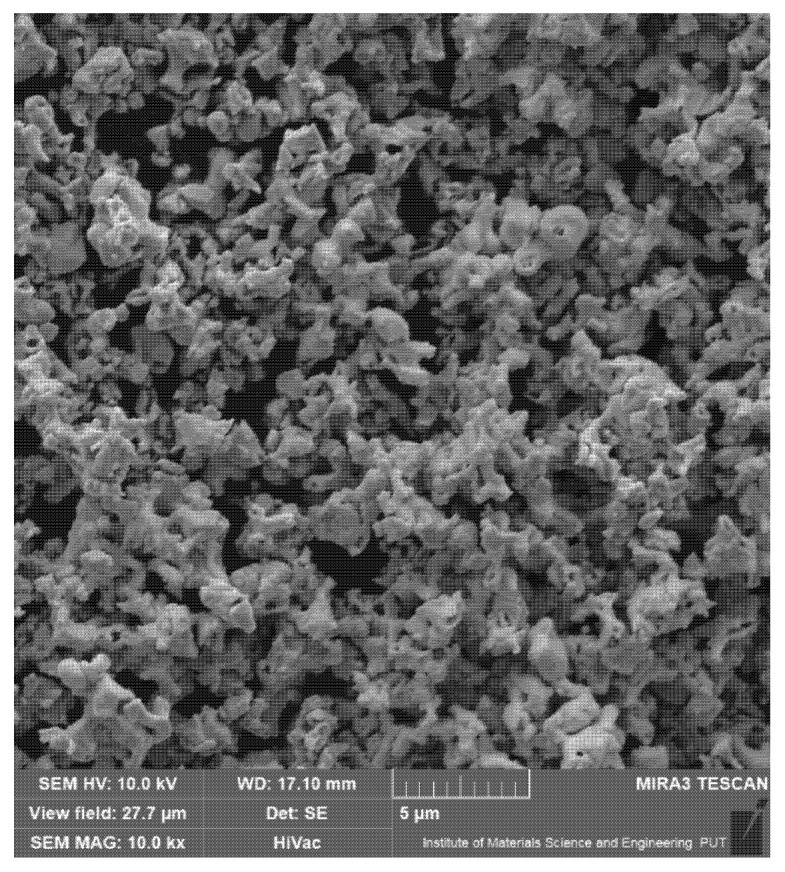
Morphology of tantalum carbide powder (APS < 6 µm).

**Figure 2:**
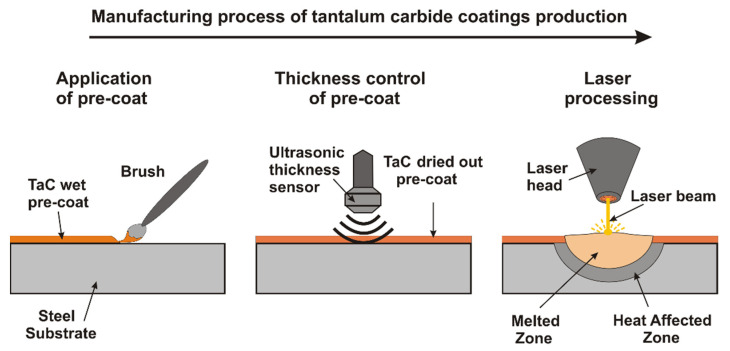
Manufacturing process of TaC coatings.

In this study specimens made of 145Cr6 tool steel in the form of tiles of 20 × 20 × 8 mm were used as the substrate. A chemical composition of this steel is presented in Table 1 and is in accordance with the manufacturer’s data delivered on the certificate. The morphology of tantalum carbide powder (shape and size) was observed by means of scanning electron microscopy and is presented in Figure 1. The average particle size (APS) was less than 6 µm. The purity of powder used in this study was 99.9% and the presented parameters were in accordance with the producer data (Sigma-Aldrich, Saint Louis, MO, USA). Prior to laser processing the pre-coatings in the form of paste were applied on the steel substrate with a brush (Figure 2). The composition of the prepared pre-coatings was as follows: tantalum carbide powder, adhesive material in the form of sodium water glass as well as distilled water. The amount of powder was selected in a weight ratio while the water glass and distilled water were adjusted so that the applied pre-coat had adequate adhesion to the steel substrate. Paste was prepared using 10 g tantalum carbide, 3 mL water glass and 3 mL distilled water. Three thicknesses of pre-coatings were applied on steel substrate: 30 µm, 60 µm, and 90 µm respectively. The thickness of the coatings was measured by a PosiTector^®^ 6000 Advance ultrasonic sensor (DeFelsko, New York, NY, USA). After pre-coatings dried, specimens were subjected to laser processing.

Laser processing was performed using TruDiode 3006 diode laser (TRUMPF, Ditzingen, Germany) with a rated power of 3 kW. During the process three different laser beam powers: 500 W, 800 W and 1100 W were applied. Laser beam diameter was 1 mm. Laser beam scanning speed for all specimens was constant at 3 m/min.

Microstructure observations were carried out using MIRA3 scanning electron microscope (TESCAN, Brno, Czech Republic) on cross-sections perpendicular to the TaC coatings produced. Prior to observation all specimens were ground using papers with grit from 80 to 2000, then polished using diamond paste and aluminum oxide, and finally etched in 5% HNO_3_ solution for 45 s. Scanning electron microscope (SEM) was equipped with an EDS-UltimMax energy dispersive spectrometer (Oxford Instruments, High Wycombe, UK) and Aztec Energy Live Standard software. The FM-810 microhardness tester (Future-Tech, Kawasaki, Japan) equipped with FT-Zero automatic indentation measuring software was used. Microhardness tests were made using indentation load of 50 g, while loading time was 15 s.

## 3. Results

### 3.1. Microstructure

**Figure 3:**
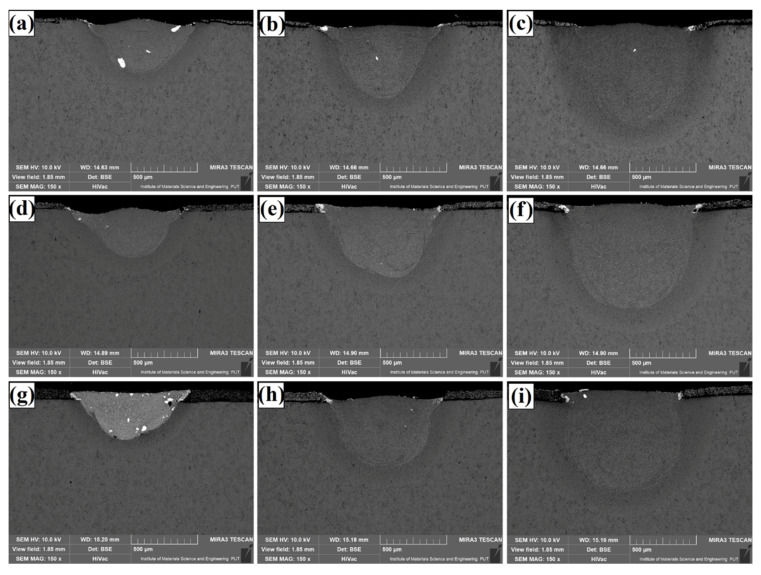
Microstructures of Fe/TaC coatings produced using 500 W, 800 W and 1100 W respectively and pre-coat with thickness of: 30 µm (**a**–**c**), 60 µm (**d**–**f**) and 90 µm (**g**–**i**).

Figure 3 shows a view of TaC composite coatings produced using three laser beam powers and different thicknesses of the initial coatings: 30 µm (Figure 3a–c), 60 µm (Figure 3d–f) and 90 µm (Figure 3g–i). The effect of laser beam power on dimensions of laser tracks is clearly visible. As laser beam power increases, so laser track dimensions increase. As a result of laser beam interaction three characteristic areas can be distinguished. The first is the remelted zone, which was formed as a result of remelting of the tantalum carbide pre-coating with the steel substrate. The second one is the heat-affected zone, while the third one is the substrate with unchanged structure. The thickness of the pre-coatings produced had little effect on dimensional changes of laser tracks. Only at the lowest laser power (500 W) it was found that increasing pre-coating thickness reduces the thickness of the produced coating. For the initial coat thickness (30 µm), TaC coating thickness produced on the steel was approximately 400 µm, while increasing the pre-coating thickness to 90 µm resulted in a reduction in the thickness of the final coating to 350 µm. This was due to the need to provide more heat to remelt the pre-coating itself. The laser beam then failed to melt a greater amount of the substrate. The use of higher power of the laser beam significantly reduced this effect. The use of a higher power of the laser beam (800 W and 1100 W) resulted in a significant remelting of the TaC particles, which resulted in the failure to obtain a typical character of a composite coating. Throughout the thickness of the coating, a uniform structure is observed, with interspersed visible white spots. These white areas are non-remelted TaC powder clusters and their presence is due to the fact that it was difficult to make a paste from such a fine grain of powder. This powder stuck together, which resulted in the formation of lumps that did not dissolve under the laser beam. The melting point of vanadium carbide is 3880 °C, hence large clusters of the material were not remelted at a fairly fast feed of the laser beam. Fe/TaC coatings produced at low power of the laser beam (500 W) look completely different. Here, the key parameter was the thickness of the prepared pre-coating. At the thickness of 30 µm and 60 µm, the laser beam caused remelting of primary TaC particles, whereas when the pre-coating thickness was 90 µm, the presence of a typical composite coating, in which the matrix is iron alloy and the phase reinforcing TaC carbide, was found. The study described in this paper was carried out in order to establish parameters for production of Fe/TaC coatings with a typical composite structure similar to that which the authors of [2] managed to produce on other materials. Microstructures of individual cases are described further in the paper. The advantage of all the produced coatings is their very good metallurgical bond with the steel substrate with a very small amount of porosity and no cracks. The main problem in producing this type of coating is the problem of applying pre-coating and adequately producing a homogeneous paste. Table 2 shows the dimensions of the obtained laser tracks. It shows the average thickness values measured along the axis of the laser track in the 10 samples produced in the tests for each of the parameters. The accuracy of pre-coating application had a tremendous influence on the size of laser tracks. The table demonstrates that the final coating thickness, including the heat-affected zone, was very similar and was mainly related to laser beam power. Where the pre-coating thickness was small, the heat affected zone was greater, and where the pre-coating thickness was large, the heat affected zone was small. For example, Fe/TaC coatings produced with the use of a laser beam power of 800 W had the final thicknesses: 685 µm (30 µm of pre-coating), 694 µm (60 µm of pre-coating) and 711 µm (90 µm of pre-coating). These thicknesses were therefore very similar.

**Figure 4:**
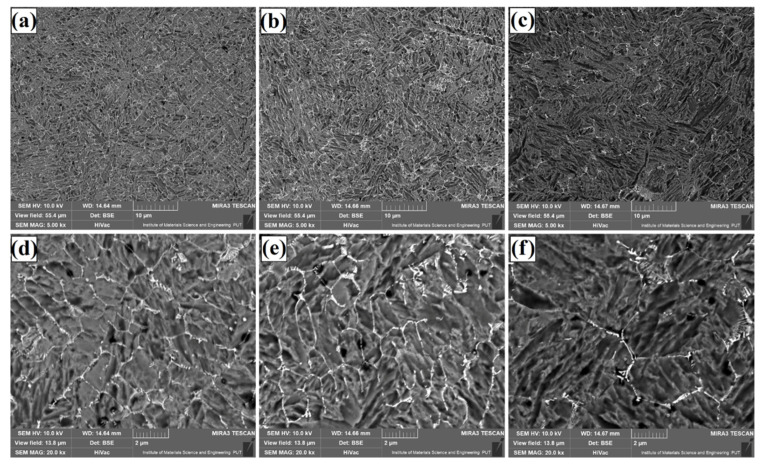
Subsurface area of the Fe/TaC coatings produced on tool steel using 30 µm thick pre-coat and laser beam power of 500 W (**a**,**d**), 800 W (**b**,**e**), 1100 W (**c**,**f**).

**Figure 5:**
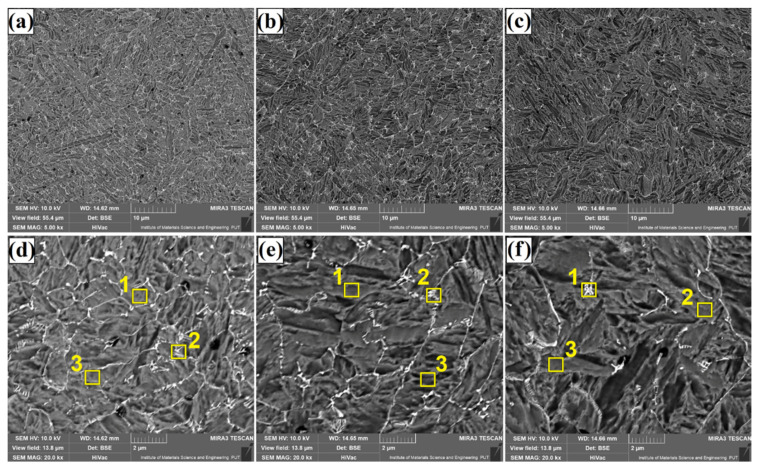
Middle area of the Fe/TaC coatings produced on tool steel using 30 µm thick pre-coat and laser beam power of 500 W (**a**,**d**), 800 W (**b**,**e**), 1100 W (**c**,**f**).

**Figure 6:**
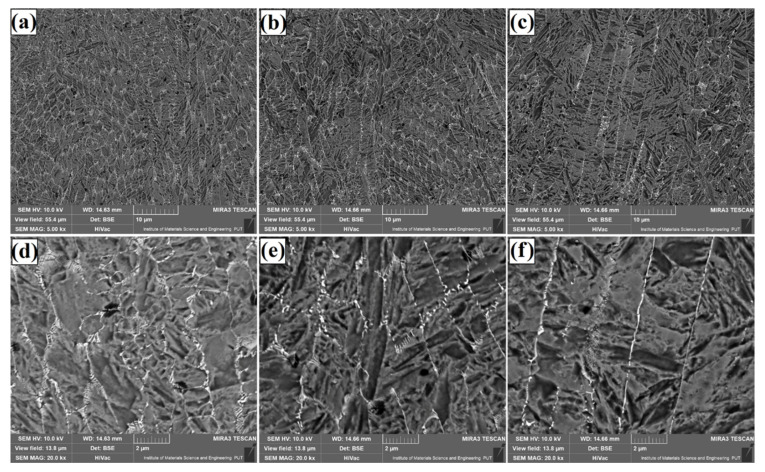
Near-substrate area of the Fe/TaC coatings produced on tool steel using 30 µm thick pre-coat and laser beam power of 500 W (**a**,**d**), 800 W (**b**,**e**), 1100 W (**c**,**f**).

Figure 4, Figure 5 and Figure 6 show the microstructure of Fe/TaC coatings produced by laser processing of a 30 µm TaC pre-coating. Characteristic images for three areas of the remelted zone are presented in turn for each laser beam power. The subsurface area (Figure 4), the middle part of the coating (Figure 5), and the area near the steel substrate (Figure 6) were analyzed. The microstructure consists of a matrix composed of Feγ dendrites surrounded by an interdendritic eutectic lattice of complex carbides formed at grain boundaries. It was found that the size and proportion of the carbide lattice decreased with increasing laser beam power. This was due to an increase in iron proportion in the coating as compared to tantalum carbide. At the highest laser beam power (1100 W), a residual amount of carbide mesh can be observed. The microstructure throughout lower, middle and upper regions of the laser track is uniform. No primary tantalum carbide particles were found anywhere. Therefore, it can be concluded that the parameters used resulted in a complete remelting of the TaC powder and the separation of secondary carbides. The spots where powder agglomerations were formed were not analyzed, as these areas were not characteristic for the entire coating obtained.

**Figure 7:**
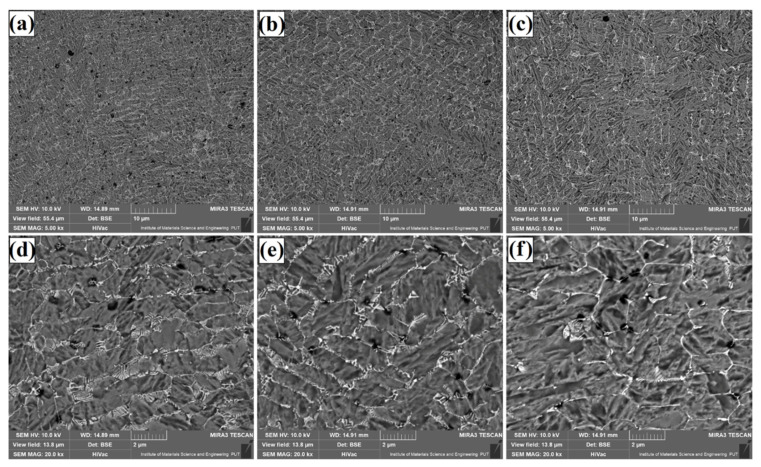
Subsurface area of the Fe/TaC coatings produced on tool steel using 60 µm thick pre-coat and laser beam power of 500 W (**a**,**d**), 800 W (**b**,**e**), 1100 W (**c**,**f**).

**Figure 8:**
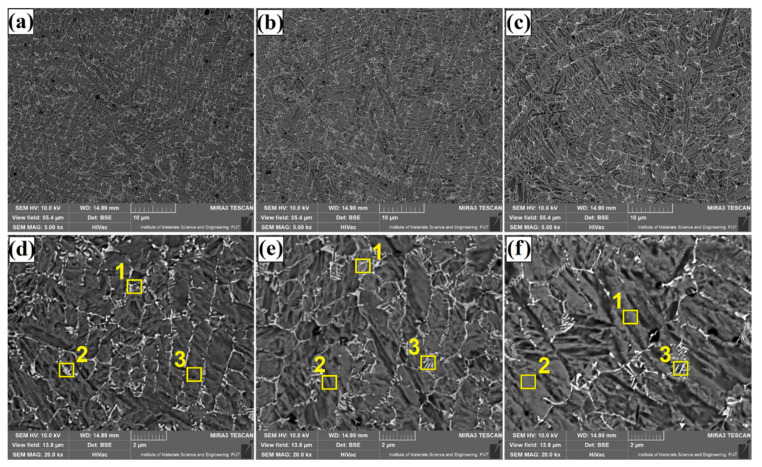
Middle area of the Fe/TaC coatings produced on tool steel using 60 µm thick pre-coat and laser beam power of 500 W (**a**,**d**), 800 W (**b**,**e**), 1100 W (**c**,**f**).

**Figure 9:**
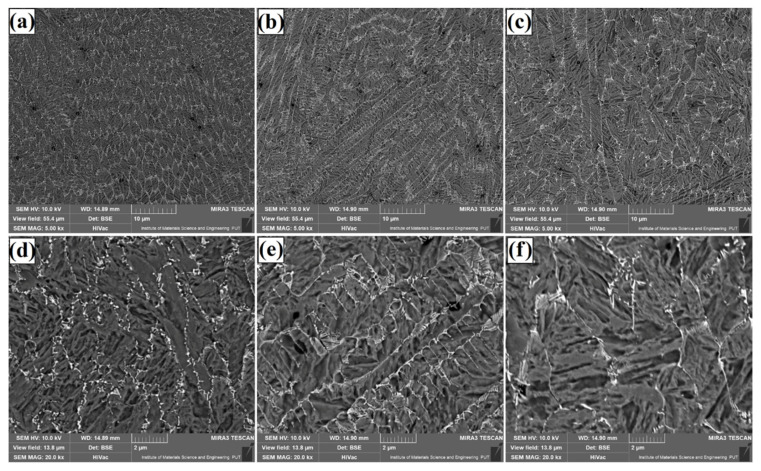
Near-substrate area of the Fe/TaC coatings produced on tool steel using 30 µm thick pre-coat and laser beam power of 500 W (**a**,**d**), 800 W (**b**,**e**), 1100 W (**c**,**f**).

The microstructure of Fe/TaC coatings produced by laser processing of the 60 µm thick TaC pre-coating is shown for each laser beam power in the subsurface area (Figure 7), the middle part of the coating (Figure 8), and the area near the steel substrate (Figure 9). A micro-structure that these coatings had was very similar to that obtained with a pre-coating thickness of 30 µm. The resulting eutectic was in the form of a mesh containing secondary carbides. However, an increase in the amount of eutectic was found due to the introduction of a larger amount of tantalum carbide into the substrate. There is also a small amount of microporosity that is formed in the matrix region. Such a property does not have to be a disadvantage. This structure may have a positive effect on self-lubricating properties of the coatings. Then it will be possible to obtain coatings that will have a hard mesh of carbides and at the same time will be less susceptible to wear due to the release of lubricant which can enrich the coating. The microstructure was even: only the direction of the carbide mesh was changed, which can be explained by the direction of solidification and mixing of the material by Marangoni forces. The structure obtained is of a composite nature, but its carbides are also secondary carbides.

**Figure 10:**
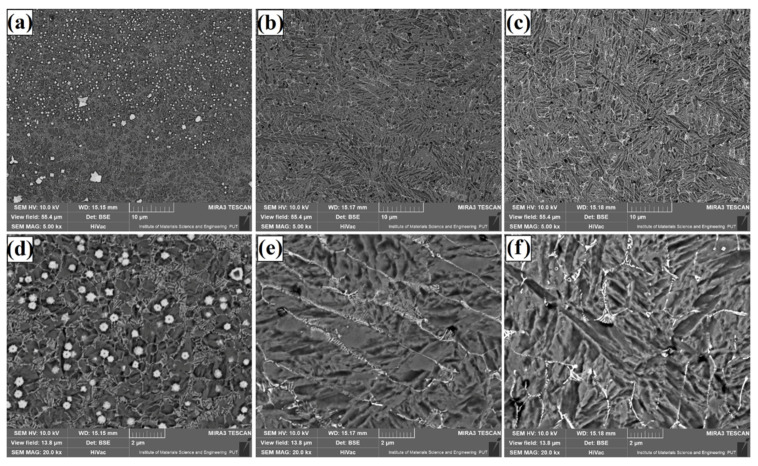
Subsurface area of the Fe/TaC coatings produced on tool steel using 90 µm thick pre-coat and laser beam power of 500 W (**a**,**d**), 800 W (**b**,**e**), 1100 W (**c**,**f**).

**Figure 11:**
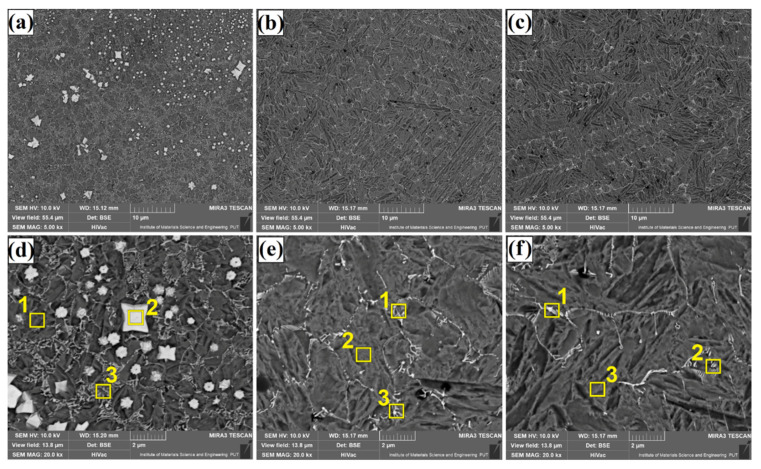
Middle area of the Fe/TaC coatings produced on tool steel using 90 µm thick pre-coat and laser beam power of 500 W (**a**,**d**), 800 W (**b**,**e**), 1100 W (**c**,**f**).

**Figure 12:**
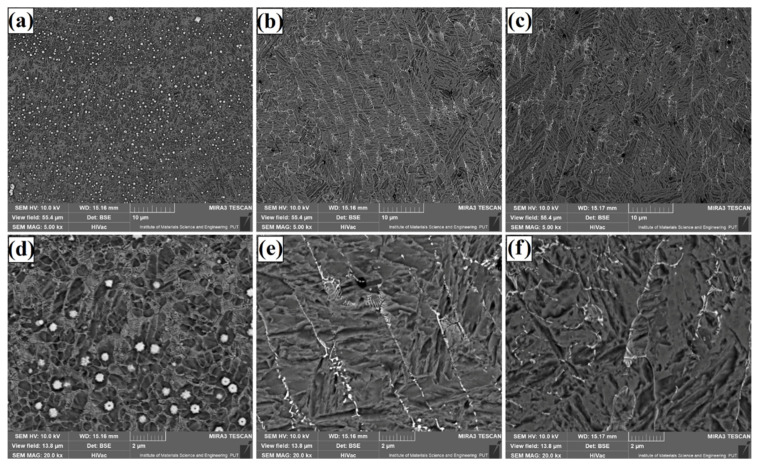
Near-substrate area of the Fe/TaC coatings produced on tool steel using 30 µm thick pre-coat and laser beam power of 500 W (**a**,**d**), 800 W (**b**,**e**), 1100 W (**c**,**f**).

The microstructure of Fe/TaC coatings produced by laser processing of a 90 µm thick TaC pre-coating is shown in Figure 9, Figure 10 and Figure 11. As for other thicknesses, the same test results presentation scheme was adopted, i.e., Figure 9 shows a sub-surface area, Figure 10—the area covering the central part of the coating, and Figure 11—the area next to the tool steel substrate. Analysis of the obtained microstructures enables division of the coatings into two types. The first type are coatings of the same nature as those obtained with a pre-coating thickness of 30 µm and 60 µm. These are coatings produced at higher powers of the laser beam, i.e., 800 W (Figure 10b,e, Figure 11b,e and Figure 12b,e) and 1100 W (Figure 10c,f, Figure 11c,f and Figure 12c,f). Their structure is a carbide eutectic in the form of a mesh. A completely different microstructure was obtained for Fe/TaC coatings produced with a laser beam power of 500 W. In all areas of this coating, the presence of both carbide eutectic and primary tantalum carbides, which were not remelted or only partially remelted, was found. It goes to show that the applied power allowed for the production of a coating of a typical composite character, where the carbides are embedded in a steel matrix enriched with carbon and tantalum. The coating formation process was strongly related to TaC particle sizes. The smallest particles of the powder remelted completely and in the process of crystallization separated at grain boundaries, creating a carbide eutectic in the form of a mesh. Slightly larger particles of tantalum carbide powder, which partially remelted, formed characteristic shapes of petals or rosettes around them. On the other hand, the largest TaC particles formed carbides with symmetrical polygonal shapes. In all the cases, high-melting primary carbides became the nucleus in the crystallization process.

### 3.2. Chemcial Composition

**Figure 13:**
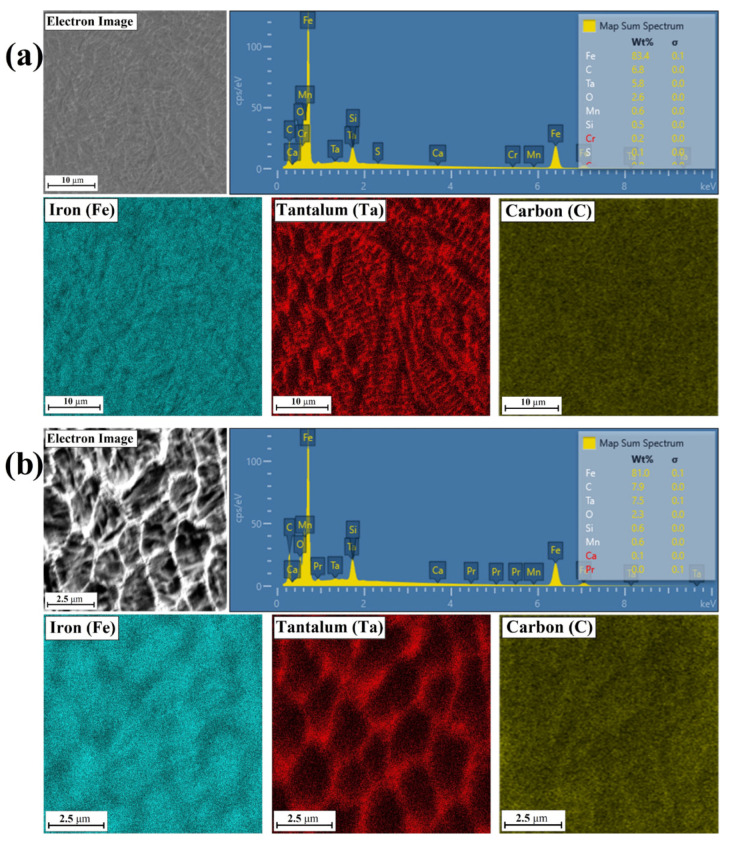
Chemical composition of middle area of the Fe/TaC coatings produced on tool steel using 30 µm thick pre-coat and laser beam power of 500 W (**a**), enlarging the examined area (**b**).

**Figure 14:**
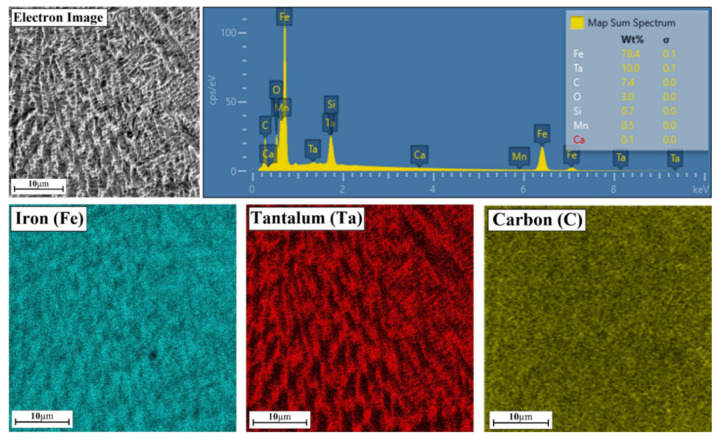
Chemical composition of middle area of the Fe/TaC coatings produced on tool steel using 60 µm thick pre-coat and laser beam power of 500 W.

**Figure 15:**
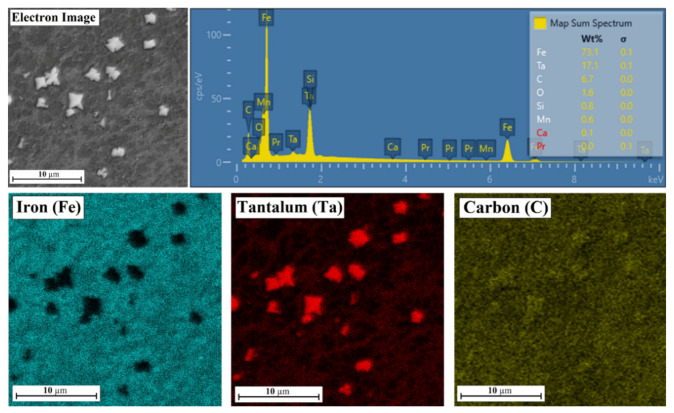
Chemical composition of middle area of the Fe/TaC coatings produced on tool steel using 90 µm thick pre-coat and laser beam power of 500 W.

**Figure 16:**
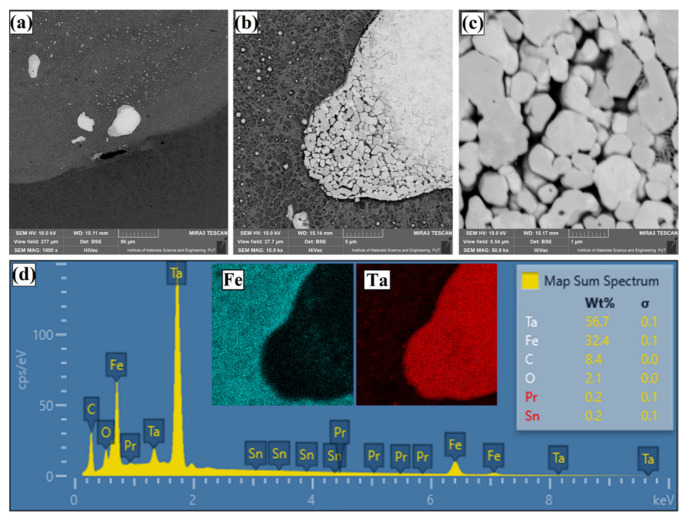
Microstructure and chemical composition of near-substrate area of the Fe/TaC coatings produced on tool steel using 90 µm thick pre-coat and laser beam power of 500 W: the border of melted zone and steel substrate (**a**), view of agglomerated and unmelted TaC powder particles (**b**), enlargement of the unmelted TaC powder particles (**c**), EDS mapping of agglomerated TaC particles (**d**).

Figure 13, Figure 14, Figure 15 and Figure 16 show the results of chemical composition tests using the EDS method, both in the form of exemplary spectra as well as maps of chemical elements. On the other hand, Table 3 shows measurement results in individual measurement points for middle area of laser tracks. The places of measurements are marked with yellow squares in Figure 5, Figure 8 and Figure 11. In order to compare the influence of pre-coating thickness on chemical composition, only Fe/TaC coatings produced with the same laser beam power of 500 W and in the central part of the remelted zone were analyzed. A Fe/TaC coating produced with a 30 µm pre-coating thickness is shown in Figure 13. Both smaller (Figure 13a) and higher magnifications (Figure 13b) clearly demonstrate the distribution of tantalum in the coating. This confirms that the white mesh visible on the microstructure corresponds to the tantalum carbides released at grain boundaries.

The maps for the Fe/TaC coating produced with a pre-coating thickness of 60 µm look very similar (Figure 14). A tantalum carbide mesh is also visible. EDS point analysis showed a greater proportion of both tantalum and carbon. This proves that there is more tantalum carbide in the mesh formed.

A Fe/TaC coating produced with a 30 µm pre-coating thickness is shown in Figure 13. Clearly, the bright irregular particles are TaC primary carbides. No iron was found in their places of occurrence, but only tantalum and an increased intensity of carbon. In addition, the presence of tantalum was also found in the matrix.

Figure 16 shows the microstructure within the TaC powder clusters. The problem of proper preparation of the paste resulted in the formation of clusters of tantalum carbide particles, which can be deemed uncharacteristic of the coating. In the microstructure of the coating, these clusters are visible as bright areas (Figure 16a). Their formation was not intended and when extending the study to include multiple tracks, special attention should be paid to the preparation of the paste intended for creation of the pre-coating. After enlarging the area containing the clusters, smaller and larger grains of TaC powder are clearly visible (Figure 16b,c). The presence of primary TaC particles is confirmed by the EDS maps and the attached spectrum (Figure 16d).

### 3.3. Microhardness

**Figure 17:**
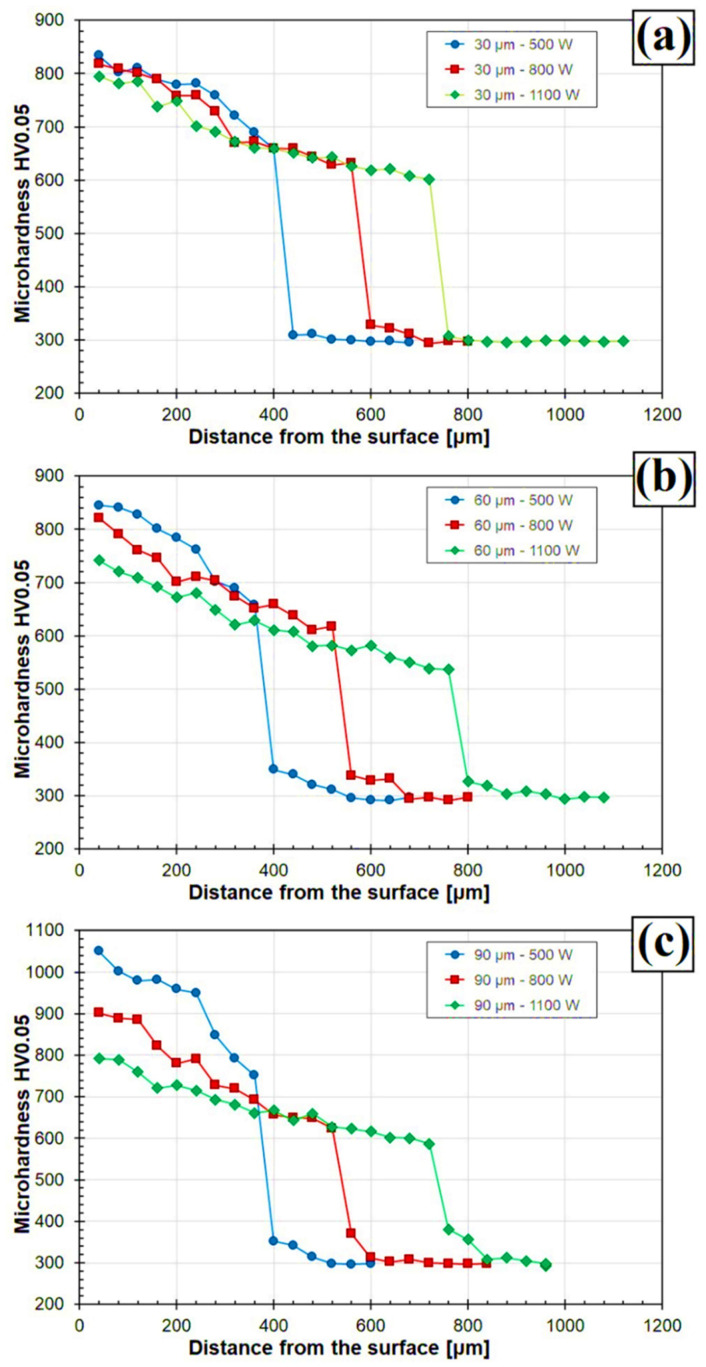
Microhardness profiles for Fe/TaC coatings produced using laser beam power 500 W, 800 W, 1100 W and applied pre-coat with thickness of: 30 µm (**a**), 60 µm (**b**), 90 µm (**c**).

Figure 17a–c shows the results of microhardness tests of composite Fe/TaC coatings produced by laser processing of paste form TaC initial coatings. A significant influence of the initial coating thickness on the obtained hardness was found: the thicker the pre-coating, the greater the hardness of coatings produced. This was due to the delivery of a greater amount of hard TaC particles. The highest hardness (approximately 1050 HV 0.05) was obtained for the Fe/TaC coating produced with a 90 μm thick pre-coating and a 500 W laser beam power. It was also observed that with increasing laser beam power, hardness decreased, which is due to greater mixing of TaC particles with the substrate steel and their remelting. The TaC particles that were remelted separated in the form of secondary complex carbides of lower hardness. In the coatings produced at high power, the proportion of iron from the substrate also increased, which resulted in reduced hardness. It is worth noting that, as far as it was possible, all hardness measurements were carried out in the axis of the track in such a way as to ignore the hard primary carbides. This proves that for TaC coatings, which retained a typically composite character (90 µm), total hardness is higher. Only the matrix, i.e., the areas between visible carbides, was measured, and it should be mentioned that tantalum carbide itself has a hardness of up to 2000 HV. It is therefore important that subsequent studies should focus on parameters that will enable production of a similar coating. As the produced coatings moved away from the surface, their hardness decreased as a result of increasing amount of iron in the coating. In the case of coatings produced with the smallest pre-coating thickness, the differences in hardness for coatings produced at different powers of the laser beam were very small (Figure 17a). Along with increasing pre-coating thickness, a greater laser beam power influence on hardness was observed, which was most noticeable for the coatings produced with a 90 µm pre-coating (Figure 17c). For all Fe/TaC coatings produced, a mild surface-to-substrate hardness gradient was observed, which is favorable in terms of stress distribution in possible industrial applications and operation.

## 4. Conclusions

The main conclusions of this study are as follows:

1. With laser processing it is possible to produce Fe/TaC metal matrix composite coatings in which the matrix is the iron from a steel substrate enriched with tantalum, and the reinforcing phase are primary TaC tantalum carbide particles.
2. Pre-coating thickness and laser beam power have a significant influence on the properties of metal matrix composite Fe/TaC coating. Application of an excessively thin pre-coating or the use of too high laser beam power leads to complete remelting of primary carbides as well as to increase in the iron content in newly formed coatings.
3. A complete remelting of primary TaC particles leads to formation of a carbide eutectic in the form of a mesh at grain boundaries.
4. The formation of a typical composite coating is also dependent on the size of the primary TaC particles introduced in laser processing. The smallest powder particles melt completely and larger particles partially melt to form structures in the form of flakes, rosettes or polygons.
5. The amount of TaC primary carbides introduced into the steel surface has a significant impact on the microhardness of obtained Fe/TaC coatings. Furthermore, the primary carbide particles cannot be completely melted because this reduces the general hardness of the coating.

## Figures and Tables

**Table 1:** Chemical composition of steel used (wt.%).

C	Mn	Si	P	S	Cr	V
1.35	0.60	0.30	0.02	0.02	1.45	0.20

**Table 2:** Thickness of laser tracks (MZ—melted zone, HAZ—heat affected zone, TT—total thickness).

Fe/TaC Coatings	MZ (µm)	HAZ (µm)	TT (µm)
Pre-coat 30 µm, 500 W	401	162	563
Pre-coat 30 µm, 800 W	552	133	685
Pre-coat 30 µm, 1100 W	763	229	992
Pre-coat 60 µm, 500 W	371	157	528
Pre-coat 60 µm, 800 W	543	151	694
Pre-coat 60 µm, 1100 W	760	205	965
Pre-coat 90 µm, 500 W	349	122	471
Pre-coat 90 µm, 800 W	542	169	711
Pre-coat 90 µm, 1100 W	751	205	956

**Table 3:** Chemical composition (EDS) of middle area of Fe/TaC coatings obtained during laser processing of TaC pre-coat.

Fe/TaC Coating	Number of Measurement	Ta [wt.%]	C [wt.%]	Fe [wt.%]
Pre-coat 30 µm, 500 W	1	5.1	8.0	86.9
	2	17.9	9.2	72.9
	3	6.3	9.0	84.7
Pre-coat 30 µm, 800 W	1	5.5	15.5	79.0
	2	22.8	15.3	61.9
	3	6.9	13.9	79.2
Pre-coat 30 µm, 1100 W	1	18.1	9.7	72.2
	2	1.7	9.4	88.9
	3	2.1	8.7	89.2
Pre-coat 60 µm, 500 W	1	26.2	10.1	63.7
	2	24.6	11.8	63.6
	3	2.9	9.1	88.0
Pre-coat 60 µm, 800 W	1	26.7	10.5	62.8
	2	4.7	7.5	87.8
	3	26.5	9.8	63.7
Pre-coat 60 µm, 1100 W	1	4.3	11.0	84.7
	2	3.8	9.3	86.9
	3	22.9	11.4	65.7
Pre-coat 90 µm, 500 W	1	17.0	9.8	73.2
	2	63.5	8.7	27.8
	3	26.3	11.4	62.3
Pre-coat 90 µm, 800 W	1	21.7	16.3	62.0
	2	2.9	16.5	80.6
	3	20.7	17.7	61.6
Pre-coat 90 µm, 1100 W	1	19.4	14.8	65.8
	2	22.9	17.2	59.9
	3	2.9	16.0	81.1

## Data Availability

Data available on request.
